# Inverse poroelasticity as a fundamental mechanism in biomechanics and mechanobiology

**DOI:** 10.1038/s41467-017-00801-3

**Published:** 2017-10-17

**Authors:** Alexander E. Ehret, Kevin Bircher, Alberto Stracuzzi, Vita Marina, Manuel Zündel, Edoardo Mazza

**Affiliations:** 1 ETH Zurich, Institute for Mechanical Systems, Leonhardstrasse 21, 8092 Zurich, Switzerland; 2 Empa, Swiss Federal Laboratories for Materials Science and Technology, Überlandstrasse 129, 8600 Dübendorf, Switzerland

## Abstract

Understanding the mechanisms of deformation of biological materials is important for improved diagnosis and therapy, fundamental investigations in mechanobiology, and applications in tissue engineering. Here we demonstrate the essential role of interstitial fluid mobility in determining the mechanical properties of soft tissues. Opposite to the behavior expected for a poroelastic material, the tissue volume of different collagenous membranes is observed to strongly decrease with tensile loading. Inverse poroelasticity governs monotonic and cyclic responses of soft biomembranes, and induces chemo-mechanical coupling, such that tensile forces are modulated by the chemical potential of the interstitial fluid. Correspondingly, the osmotic pressure varies with mechanical loads, thus providing an effective mechanism for mechanotransduction. Water mobility determines the tissue’s ability to adapt to deformation through compaction and dilation of the collagen fiber network. In the near field of defects this mechanism activates the reversible formation of reinforcing collagen structures which effectively avoid propagation of cracks.

## Abstract (web summary)

How soft tissues respond to mechanical load is essential to their biological function. Here, the authors discover that – contrary to predictions of poroelasticity – fluid mobility in collagenous tissues induces drastic volume decrease with tensile loading and pronounced chemo-mechanical coupling.

## Introduction

In terms of weight and volume, water is indisputably the major component of soft biological tissues. The mechanical property most commonly associated with the presence of water is near volume constancy, i.e., incompressibility, based on the fact that the compression modulus of water is orders of magnitude larger than the distortional stiffness of tissues^1^. The vast majority of biomechanical studies on soft collagenous tissues is based on this assumption^2^, typically without experimental verification of its validity. While this assumption implies that all the interstitial fluid is immobile and bound to the tissue, other biomechanical analyses build, vice-versa, on the mobility of the liquid phase in a porous matrix, driven by spatial pressure gradients. The role of water in such biphasic or porous media representations thus lies in that it furnishes the tissue with time-dependent characteristics governed by the chemical and physical properties of fluid and solid phase^3^. The hydrostatic pressure in a material that is under uniaxial (UA) tensile stress *σ* is −*σ*/3, hence negative, and Biot’s theory of poroelasticity^4^ tells us that such negative pressure should lead to an increase of volume under tensile loading. For a saturated porous medium, this indicates *in*flux of water. In contrast to both conceptions, negative compressibility^5^, documented by Poisson’s ratios larger than 0.5 and volume loss during UA tensile loading, has been reported for tendons and ligaments^6–8^, human amnion^9, 10^, samples prepared from articular cartilage^11^, as well as collagen and fibrin-based hydrogels^12–15^. This entails *ef*flux of water^14, 16^ for negative hydrostatic pressure, and we refer to this behavior as inverse poroelasticity. The corresponding volume changes reported for tendons and ligaments^6–8^ are comparably small, and substantial volume reductions in cartilage^11^ were elicited only by large elongations.

Here we demonstrate that various soft collagenous membranes experience very large volume reduction already for moderate in-plane tensile loading, that these volume changes are reversible, and that they are a collective result of the particular arrangement and kinematics of the collagen network and the inherent chemoelasticity of soft biological tissues. While the important role of water in creating residual stresses in tissues by swelling has been identified^17^, and its impact on the mechanics of collagen on a molecular scale was recently highlighted^18^, the present data demonstrate the key role of water mobility in determining the tissue response, and identify an essential deformation mechanism with far-reaching implications for the biomechanics and mechanobiology of soft biological tissues. We show this on the basis of UA and biaxial tensile experiments on human amniotic membrane (hAM), bovine liver (Glisson’s) capsule (bGC) and porcine pericardium (pPC), i.e., three different types of biological tissues. Monotonic and cyclic loading protocols are applied in macroscopic tests and in situ, in a multiphoton microscope (MPM), allowing for a quantitative analysis of the 3D kinematics of the tissue associated with each loading condition. Drastic volume changes are associated with strong variations in water content. As a consequence, the osmotic pressure varies with mechanical loads and, vice-versa, the level of tension for a given deformation depends on the chemical potential of the interstitial fluid. The observed chemo-mechanical coupling is rationalized through corresponding biphasic constitutive models and is proposed as an effective mechanism for mechanotransduction. Inverse poroelasticity and the associated compaction and dilation of the collagen fiber network provide soft tissues with unique mechanical properties, such as high compliance for moderate stretching and strong resistance to larger non-physiological deformations. MPM observations of the near field of crack-like defects demonstrate that these mechanisms induce a localized reversible densification of collagen protecting the tissue from crack propagation.

## Results

### Volume changes in soft collagenous membranes

**Fig. 1:**
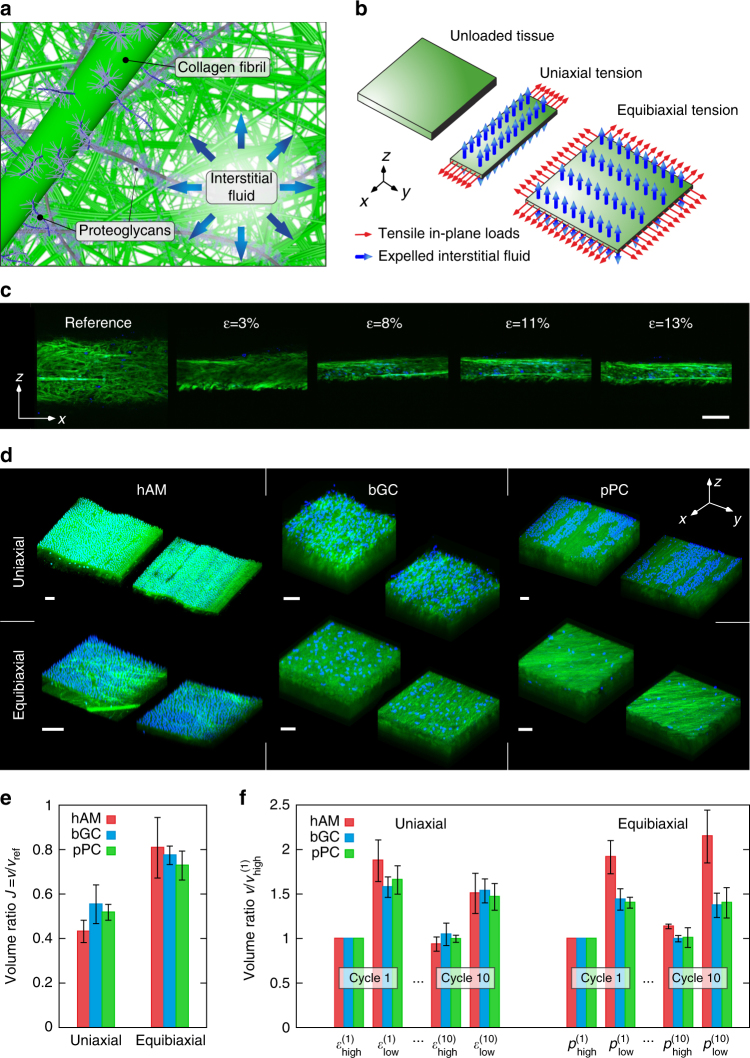
Volume changes in soft collagenous membranes. **a** Illustration of extracellular matrix components mainly responsible for the mechanical behavior of soft collagenous tissues: collagen fibrils and fibers, proteoglycans carrying negative charges and interstitial fluid. **b** Schematics of the response to in-plane mechanical forces: unloaded and deformed tissues in uniaxial (UA) and equibiaxial (EB) tension. **c** Thickness reduction of bovine Glisson’s capsule (bGC) sample in UA tension with increasing applied elongation ε; cross-sectional multiphoton microscopy (MPM) images of collagen (*green*) and stained cell nuclei (*blue*), taken close to the lateral edge of a sample. *Scale bar*: 100 μm. **d** MPM stacks in reference and deformed state demonstrate strong thickness reduction at ~10% in-plane elongation. *Scale bars*: 50 μm. **e** Volume reduction *J* = *λ*_1_ *λ*_2_ *λ*_3_ in UA and EB tension from combined macroscopic (human amnion (hAM): *n* = 9, bGC: *n* = 5, porcine pericardium (pPC): *n* = 5) and MPM (hAM: *n*=3, bGC: *n* = 3, pPC: *n* = 3) data. Reported is mean and standard deviation (*error bar*). **f** Volume change during cyclic loading (10 cycles) in UA (strain states *ε*_high_ = 12%, *ε*_low_ = 0%) and EB (inflation pressure states *p*_high_ = 4 kPa (hAM)/55 kPa (bGC, pPC), *p*_low_ = 0.1 kPa) configurations. The data are normalized with respect to the volume at *ε*_high_ and *p*_high_ in the first cycle. Reported is mean and standard deviation (*error bar*) for hAM (macroscopic: *n* = 7, MPM: *n*_UA_ = 3, *n*_EB_ = 4), bGC (macroscopic: *n* = 5, MPM: *n* = 3) and pPC (macroscopic: *n* = 5, MPM: *n*=3). Some of the hAM data include information from refs.^10, 23, 54^

The liquid volume fraction of the considered membranes is typically >80%, the network of collagen fibers (mainly types I and III) constitutes a significant amount of tissue dry weight and no significant elastin content was reported for the bulk of these membranes^19–21^. Considered as a mechanical system, these tissues thus consist mainly of water within a network of collagen fibers, enriched with negatively charged proteoglycans (Fig. 1a). The present analysis of their deformation behavior is based on the kinematic response of membrane samples to UA tension and in-plane biaxial tension, performed in wet conditions. Figure 1b illustrates qualitatively the observed kinematic response to UA and biaxial loading common to all three membranes: large contraction perpendicular to the loading direction leads to fluid efflux and strong volume reduction upon application of tensile forces. MPM images of the lateral cross sections taken at increasing values of elongation in in situ UA tensile tests highlight the drastic reduction in thickness (Fig. 1c). To quantify volume changes in all three membranes, the in-plane state of deformation was retrieved from the displacement field of a surface pattern in macroscopic experiments^22^ (Supplementary Fig. 1a), while MPM image stacks from in situ experiments were used to determine the corresponding changes of thickness for UA and biaxial loading conditions (Fig. 1d). On the basis of these data, the apparent Poisson ratios^13, 23^ and the change in volume for monotonic tensile tests were quantified (Fig. 1e). Poisson’s ratios change with elongation and reach maxima up to 6 (Supplementary Fig. 1e, f), about twice the values reported for biopolymer gels^13, 14, 24, 25^. For moderate UA elongation, the volume decreases by up to a factor of two for all three membranes, and even four-fold when larger forces were applied (Supplementary Fig. 1d). Less, but still remarkable volume reduction was associated also with equibiaxial (EB) in-plane tension (Fig. 1e). The question arises whether the drastic volume changes are a characteristic of the very first loading step (after tissue extraction from the organism) that would accordingly not be present or, at least, much smaller in successive loading cycles. To evaluate this, tissue specimens were cyclically loaded and unloaded 10 times between two load levels, the upper one causing ~10% elongation, and the corresponding volume changes were determined. The results convincingly showed that volume loss and regain persist over the cycles, with volume changes of 50% or larger (Fig. 1f). Volume increase and decrease hence represent a characteristic feature of the deformation behavior of all three membranes under both UA and biaxial loading conditions. In terms of resistance to deformation, all tissues display the characteristic *J*-shaped tension-stretch curves^23, 26^ well known for soft biological materials (Supplementary Fig. 1b). The in-plane lateral contraction in UA tensile tests is by a factor of 5 to 10 larger than the one expected for isotropic incompressible materials^23, 26^ (Supplementary Fig. 1c) and both this contraction and the volume reduction data are highly reproducible as opposed to the typically large variability of the corresponding tension-elongation curves (Supplementary Fig. 1b–d).

Assuming water volume fractions in the range of 80%, our results suggest that more than 60% of the liquid phase is expelled from the tissue for the applied tension level, which induces an order of 10% in-plane elongation. Such a drastic level of dehydration is expected to affect the osmolarity of the tissue. Vice-versa, the osmotic pressure difference between tissue and its environment should thus influence the tension level for a given state of deformation. This chemo-mechanical coupling was investigated in a set of dedicated experiments.

### Chemo-mechanically coupled tissue response

**Fig. 2:**
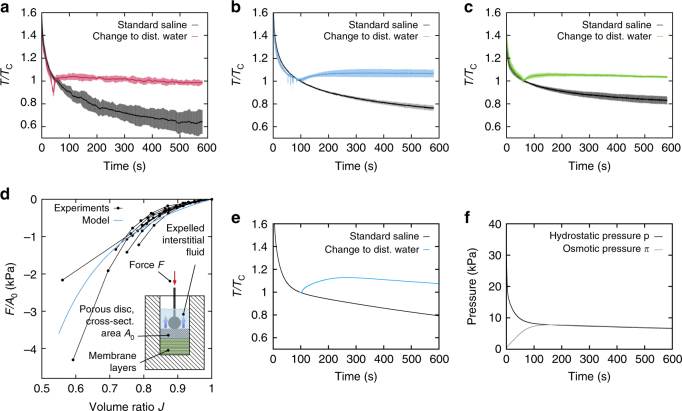
Chemo-mechanically coupled tissue response. **a**–**c** Uniaxial (UA) tension relaxation experiments on (**a**) human amnion (hAM, *n* = 3, peak force 0.2 N), (**b**) bovine Glisson’s capsule (bGC, *n* = 3, peak force 0.8 N) and (**c**) porcine pericardium (pPC, *n* = 3, peak force 0.8 N) performed in 0.9% NaCl solution without (*black*) and with bath change to distilled water (*color*). The data are presented as mean (*solid curve*) and standard deviation (*shaded area*) and normalized by the corresponding tension value (*T*_C_) at the time point of change of bath. **d** Experimentally determined relaxed pressure (force per area) vs. volume ratio *J* curves for bGC sample (*n* = 9 stacks, *black*) together with the response of the biphasic model (*blue*). *Inset*: schematic illustration of the confined compression set-up. **e** Temporal evolution of tension in simulated UA relaxation experiment for bGC without (*black*) and with (*blue*) bath change to distilled water. **f** Evolution of hydrostatic (*black*) and osmotic (*gray*) pressures in the center of the specimen, corresponding to **e** without change of bath

We had previously shown that tension reduces considerably with time in relaxation tests, i.e., upon application of a fixed level of elongation in UA tension experiments for hAM^9^ and bGC^26^. The relative reduction of tension with time was highly reproducible for the same testing protocol, despite the typically large variability among samples of biological origin. Making use of the high reproducibility of this protocol, test pieces were clamped and equilibrated in physiological saline solution, then rapidly elongated to a given target force, and kept at the corresponding length while the force signal was recorded. After a short time, however, the bath was changed to distilled water. The change to a hypotonic environment leads to a strong reduction or even reversal of the stress relaxation in all tissues (Fig. 2a–c). These results demonstrate that the mechanical response of the membranes can be actively controlled by setting the chemical potential of their environment, and reveal the significance of chemo-mechanical coupling mechanisms. To rationalize the osmosensitive relaxation characteristics of the membranes, the tissue was considered as a highly hydrated, i.e., swollen biphasic material consisting of an incompressible solid phase, mainly the network of collagen fibers, and water. Swelling occurs due to the osmotic pressure difference *π* caused by charge-independent and charge-dependent effects, which are mainly attributed to the presence of proteoglycans^27, 28^ within the collagenous matrix. In an equilibrium state, *π* equals the hydrostatic pressure *p* acting on the interstitial fluid phase, which is balanced by the mechanical response of the dilated collagen network. When either osmotic pressure or mechanical stress change, the gradient of the difference *p*−*π* drives the water through, and over the boundaries of the porous matrix, leading to a change of tissue volume. The Cauchy stress tensor ***σ*** and fluid flux vector ***q*** were thus modeled as^28–30^

1 $${\boldsymbol{\sigma}} = {{\boldsymbol{\sigma}}_{\rm{s}}} - p{\mathbf{I}},\;{\boldsymbol{q}} = - {\mathbf{k}}{\rm{grad}}\left( {p - \pi } \right),$$

where ***σ***_s_ represents the stress in the solid phase, **I** is the identity tensor and **k** denotes the deformation dependent hydraulic conductivity^31^ of the tissue. In our model ***σ***_s_ is defined based on equations representing the viscoelastic response of soft collageneous membranes^26, 32^. The hydraulic conductivity of collagenous membranes is typically in an order of^33^ 10^−14^ to 10^−12^ m^4^ N^−1^ s^−1^. For membranes of a few hundred microns thickness this leads to a fast exchange of water with time scales in the range of seconds (Fig. 2a–c). This indicates that water flow can affect typical physiological tissue deformations caused by, e.g., the cardiac cycle, breathing, fetal movements or uterine contractions. The osmotic pressure *π* increases with reducing tissue volume, which can be explained by entropic effects and the increased density of fixed negative charges present at the proteoglycans^29^, leading to a flux of mobile ions according to the Donnan equilibrium^34^. Since the parameters of these theories are largely unknown for the tested soft tissue membranes, we quantified the relation between *π* and the volume change *J* for bGC from confined compression experiments (Fig. 2d). The outcome was used to rationalize the measurements (Fig. 2e) and provided indicative results on the corresponding time histories of hydrostatic and osmotic pressure within the tissue (Fig. 2f).

Notably, both measurements and calculations (Fig. 2d, f) indicate that volume decrease is associated with variations in osmotic pressure of several kPa. Such significant changes in osmotic pressure are known to affect cell homeostasis and mitosis^35–37^. Additionally, the corresponding fluctuations in hydrostatic pressure, here up to 30 kPa (Fig. 2f), contribute to the mechanical cues arising from inverse poroelasticity.

The results in Fig. 2 further demonstrate that the osmotic pressure difference between the tissue and its environment, which depends on the proteoglycan density, and thus on the current volume of the tissue, provides a mechanism of resistance to deformation. Higher density increases the resistance to fluid outflow and decreases tissue compliance. Osmotic pressure is thus an important determinant for the biomechanics and mechanobiology of soft collagenous membranes. The other key component in determining the inverse poroelasticity of these membranes is the network of collagen fibers.

### Kinematics of collagen fiber networks

**Fig. 3:**
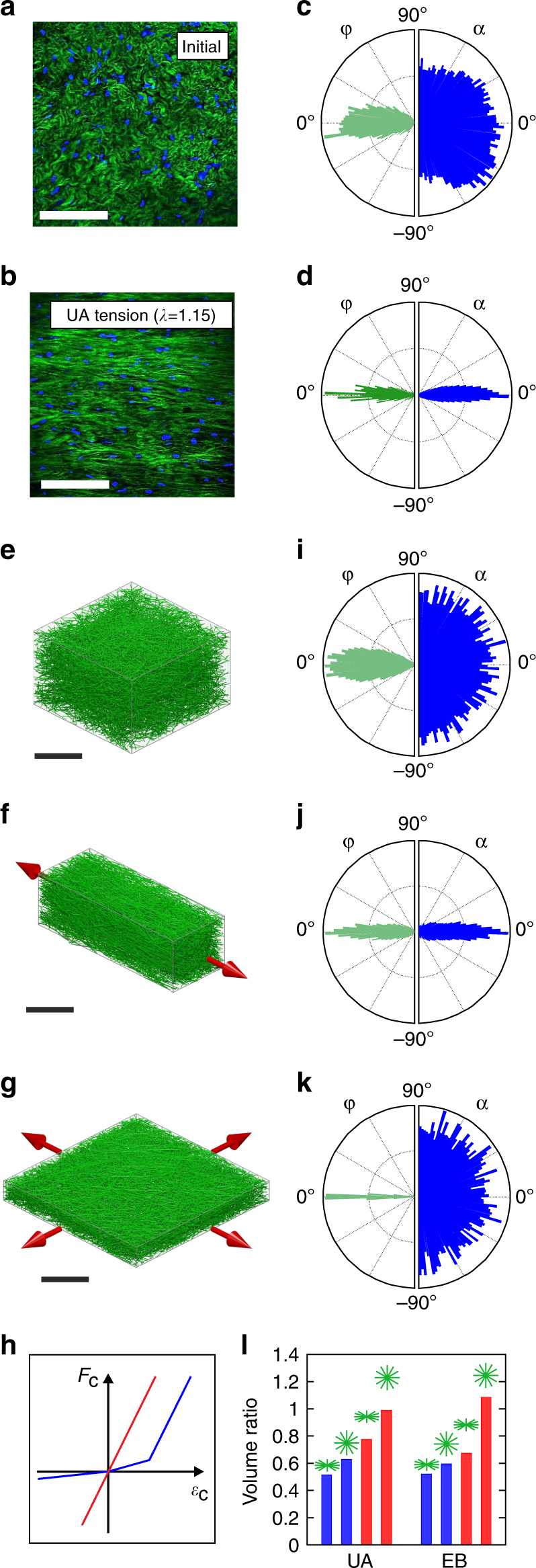
Kinematics of collagen fiber networks. **a**, **b** Multiphoton Microscopy (MPM) images of bovine Glisson’s capsule (bGC) in initial (**a**) and (**b**) deformed configuration (uniaxial (UA) elongation of ~15%, i.e. *λ*_1_≈1.15); Second harmonic generation (SHG) signal (*green*) and fluorescence of stained cell nuclei (*blue*). *Scale bar*: 100 μm. **c**, **d** Out-of-plane (*green, left*) and in-plane (*blue*, *right*) fiber orientation distribution measured in a representative bGC sample under the same conditions as for **a**, **b**. **e**–**g** Visualization of the 3D discrete network model mechanically equivalent to bGC, in initial and deformed configuration under UA and equibiaxial (EB) tension at *λ*_1_ = 1.15 (loading directions marked by *red arrows*). *Scale bar*: 100 μm. **h** Qualitative axial force vs. strain diagram of linear (*red*) and nonlinear (*blue*) fiber response. **i**–**k** Computed out-of-plane (*green*) and in-plane (*blue*) orientation distributions corresponding to **e**–**g**. **l** Volume ratios with respect to reference configuration at *λ*_1_ = 1.15, computed for different network types, with either initially oriented or isotropic fiber distribution in out-of-plane direction (*green symbol*), and either nonlinear (*blue*) or linear (*red*) fiber response

Slender collagen fibers are highly compliant in compression, due to their low bending stiffness, and gain high stiffness in tension. This key characteristic of biopolymer filaments has been previously identified to allow for large Poisson ratios^38^ in fibrous networks, and to cause the negative Poynting effect^39^ in various biopolymer gels (Supplementary Discussion). Instead of stretching, fibers in the network have the tendency to accommodate tissue elongation by rotation towards the loading direction^25^. This is the main cause of the kinematic response observed for soft collagenous membranes. Figure 3a–d illustrates the difference in tissue morphology between initial (unloaded) and deformed configuration in a UA tensile experiment on bGC. In the deformed state, fibers are consistently oriented towards the loading direction, as indicated by the angular distribution histogram (Fig. 3d). In particular, the collective reorientation within the membrane plane and the reduction of the out-of-plane inclination cause very strong lateral contractions, and volume loss. We thus hypothesized that the observed strong volume reductions at moderate elongations would be favored by the particular, transversally isotropic organization of the fibers with a small inclination with respect to the membrane plane (Fig. 3c).

To investigate this, we created a network model (cf. ref. ^25^) for the membranes (Fig. 3e, i), in which fibers are represented as discrete load bearing elements, with high stiffness in tension and low stiffness in compression (Fig. 3h). The role of the interstitial fluid in equilibrium states is represented by a compressible matrix, with initially low resistance to volume reduction that strongly increases at low volume when osmotic effects oppose water outflow. The strong contraction perpendicular to the loading direction, eliciting drastic volume changes and strong reorientation of fibers towards the loading directions, is consistently reproduced by the fiber network model for UA and biaxial loading conditions as indicated by polar plots of the fiber orientations (Fig. 3f,g,j,k). Conversely, when fibers are represented with equal stiffness in tension and compression, lateral contractility and, consequently, volume reductions are significantly lower (Fig. 3l). Likewise, calculations assuming isotropic initial fiber arrangement weaken the volume changes, and a combination of the two modifications finally determines a nearly incompressible response, and even reverses the effect slightly for EB tension (Fig. 3l). These results confirm that the early volume collapse in collagenous membranes is a collective result of both the strong nonlinearity in the response of slender fibers, which are stiff when tensioned but buckle in compression, and the transversally isotropic distribution of fibers, caused by their low out-of-plane inclination.

The interplay between fiber kinematics and osmotic effects driving water from and to the tissue leads to an effective deformation behavior which allows material to resist large stretches, but to easily adapt to moderate, physiological deformations. This adaptive compliance is realized through the recoverable process of water efflux, thus without permanent deterioration of the microstructure. We speculated that this characteristic feature of their deformation behavior is intimately related to the outstanding defect tolerance of soft biological tissues^40, 41^. To investigate this hypothesis, we analyzed the near field of defects in fetal membranes.

### Reinforcement and stress shielding in near fields of defects

**Fig. 4:**
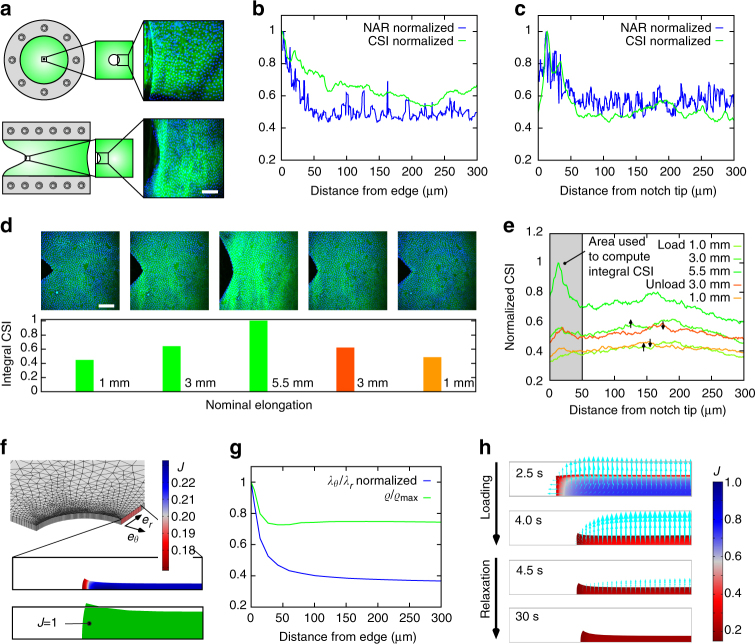
Reinforcement and stress shielding in the near field of defects. **a** Inflation and fracture testing set-ups and multiphoton microscopy (MPM) images of human amnion (hAM) samples with circular or notch-like defects. *Scale bar*: 100 μm. **b**, **c** Near fields of a circular defect at 5.6 kPa inflation pressure (**b**) and a notch at 6 mm nominal elongation (**c**). Normalized nuclear aspect ratio (NAR) and collagen signal intensity (CSI) rapidly drop to their far-field levels with distance from the defect edge/notch tip. (One representative hAm sample out of *n* = 3, respectively). **d** Reversible formation and dissolution of reinforcing structures close to the notch tip during a load cycle, quantified by normalized integral CSI over 50 μm. *Scale bar*: 100 μm. **e** Reversibility of collagen densification in near and far-field of the defect during loading and unloading. **f**–**h** Simulation results for membrane with circular hole rapidly stretched and kept at constant far-field strain of 8% (One octant shown). **f** Volume change *J* (*top*) and deformed state following from the assumption of incompressibility (*bottom*). **g** Circumferential to radial stretch ratio and density, normalized by their values at the defect edge, showing a rapid drop. **h** Efflux of water (*arrows*) and volume change *J* over time (*color bar*)

Both soft and hard biological tissues have been identified to be highly defect tolerant, and explanations based on their microstructure were proposed^41–44^. Fracture mechanics analyses of hAM, in particular, are related to the medical problem of iatrogenic premature rupture of the fetal membrane after invasive prenatal intervention^45^. We performed in situ experiments in order to analyze the local mechanisms of deformation in the near field of a defect in hAM. Biaxial tests were performed on specimens with a central, circular hole of 1mm diameter, and typical mode-I fracture test pieces^46^ with a lateral cut were tested in strip-biaxial tension (Fig. 4a), while near and far-fields of the defects were monitored. The intensity of the collagen second harmonic generation (SHG) signal (CSI) was considered as a measure of collagen compaction, and the nucleus aspect ratio^47^ (NAR) of epithelial cells, was used to identify the zone kinematically affected by the defect. The CSI is maximal in the direct vicinity of the defect and drops rapidly to a mean level comparable to that of the far-field (Fig. 4b, c). This indicates that the collagen fibrils form a dense reinforcement at the defect that effectively prevents crack propagation through the tissue and shields the remote areas (Supplementary Video). The NAR, as an indicator of strain, is likewise at maximum at the defect sites and rapidly decays towards the far-field level (Fig. 4b, c), which takes a value of about 1.25 for EB stretching before normalization, in good agreement with values reported for roundish epithelial cells^48^. Figure 4d, e illustrates that a dense collagen fiber bundle forms at the defect when load is applied and dissolves almost entirely upon unloading, thus indicating that the process leading to the local reinforcement is reversible, in agreement with the reversibility of volume changes observed for intact samples (Fig. 1f). Inverse poroelasticity and strong effective compressibility are key to enable these protective mechanisms, which would not be possible if water was immobile. This is illustrated by simulations with the biphasic model described above. A realistic value for the parameter defining the initial hydraulic conductivity in the order of 10^−14^ m^4^ N^−1^ s^−1^ was used and, for comparison, zero-permeability was assumed in a second simulation, which corresponds to the widespread assumption of incompressibility. While the reference model predicts a rapid transition of strain and volume change with increasing distance from the defect (Fig. 4f, g), the incompressible model cannot reproduce the observed compaction mechanism (Fig. 4f). Although CSI and NAR do not provide direct quantitative measures of tissue density and deformation, it is interesting to note that the reference model predicts densification and circumferential to radial stretch ratio well in line with the patterns of CSI and NAR observed in hAM (Fig. 4b, g). The simulation reveals that the densification proceeds rapidly and is even facilitated at the defect site, where efflux occurs both over the membrane face and the lateral defect surface (Fig. 4h).

## Discussion

The analysis of the 3D kinematics of biomembranes revealed deformation characteristics that contradict the common expectations based on incompressible or poroelastic theories. The tissue response can be understood as inversely poroelastic and be explained by the interplay between collagen fibers and interstitial fluid, interacting as in a tensegrity-like structure^49^: While the former provides the tensile load bearing elements, the latter carries compressive forces and prevents the collapse. The pronounced water mobility and effective compressibility of the membranes analyzed in the present work are expected to apply to a variety of soft collagenous tissues with similar structure and physiological function, though biomaterials with lower hydraulic conductivity, lower initial water content, higher density of proteoglycans, or larger non-collagenous matrix fractions (cf. ref. ^13^) may be less affected by inverse poroelasticity, and their treatment as incompressible solids in biomechanics may represent a reasonable approximation.

Large volume loss in tension, albeit counterintuitive, is not in conflict with thermodynamics for nonlinear materials (Supplementary Discussion and Supplementary Fig. 3). Negative compressibility of biopolymer hydrogels^12–14^ was attributed to strong reorientation and buckling of collagen fibers^25^, or conformational changes in fibrin^24^. The investigated soft tissue membranes stand out from other tissues with negative compressibility^6–8, 11^ as reducing their volume drastically already with moderate elongations. This is pinpointed by high Poisson’s ratios up to 6, and explained by the transversely isotropic alignment of the collagen fibers in these thin tissues, which amplifies the effects of fiber nonlinearity and reorientation. The mobility of interstitial fluid is a second key element that determines the response of tissues in physiologically relevant tensile loading states. Volume changes occur only due to the ability of water to move out of or into the tissue, allowing to accommodate to elongations or to effectively defuse a defect exposed to stress concentrations. By reflux, this mechanism of adaption is reversible, thus leading to high toughness without permanent microstructural deterioration. An additional toughening effect in porous media is related to the motion of water within the near field of the defect, as proposed for polymer gels^50^.

Our results demonstrate that moderate in-plane elongations lead to increased hydrostatic pressure (Fig. 2f), significant changes of tissue hydration and thus in the chemical potential of the interstitial fluid. Variations in hydrostatic and osmotic pressure between cell interior and their environment affect cell hemostasis (e.g., ref. ^37^) and represent fast and homogeneously distributed signals of the occurrence of mechanical loads. The chemo-mechanical coupling induced by the inverse poroelastic behavior thus suggests itself as a very effective mechanism of mechanotransduction, different from the integrin-based pathways^51^. Our results indicate that the architecture of the collagen fiber network and the amount of proteoglycans represent key features of the extracellular matrix to control the magnitude of these mechanical cues and the effectiveness of their transmission.

As another implication of inverse poroelasticity, tensile loads might cause significant hydrostatic loading of a confluent superficial cell layer lining the interface between soft collagenous tissues and their environment. In fact, as demonstrated by a corresponding simulation (Supplementary Methods and Supplementary Fig. 2), the inverse poroelastic effect elicits an outward directed rising pressure during in-plane loading. Potential “hydraulic fracture” of the cell layer is thus expected to occur during the tensile loading phase in biological membranes, in contrast to recent results for cell substrates made of polyacrylamide hydrogel, which report fracture during unloading^52^. This difference between hydrogels and biological tissues is expected to be relevant for tissue engineering applications^49^ with respect to the mechanical biocompatibility^53^ of materials for cell culture.

## Methods

### Macroscopic and in situ characterization of soft tissue membranes

Porcine hearts and bovine liver tissue were received from the local slaughterhouse short time after animal euthanasia, after inspection by a veterinarian. Membrane samples were prepared by gentle separation of the organ capsules (bGC, pPC) from the underlying tissue layers^26^. hAM was obtained from fetal membranes of patients undergoing elective term cesarean sections, according to the criteria defined in the ethical approval (Ethical Committee of the District of Zurich Stv22/06 and Stv07/07). Experiments with animal and human tissue were performed within few hours after extraction from the body. Membranes were immersed in 0.9% saline solution at room temperature at all times before and during experiments, and kept hydrated during sample preparation.

Rectangular and circular test pieces for UA and inflation (equibiaxial, EB) testing, respectively, were cut using a surgical scalpel, placed and clamped in the testing devices as described in refs.^9, 26^. Test pieces for macroscopic testing were cut to dimensions of 80 mm  × 15 mm (free length 60 mm) for UA tests and to 70 mm diameter (free diameter 50 mm) for EB tests. In-plane deformations were determined by tracking the positions of ink markers (GEOCollege Pigment Liner 0.05) on the surface of the test pieces (Supplementary Fig. 1a) recorded by a CCD camera (UA: Pike F-100B Allied Vision Technologies GmbH, Stadtroda, Germany / EB: Dragonfly 2, Point Grey, Richmond, Canada). Images were analyzed with a custom-built algorithm^22^ to extract local displacement fields in a preselected central region of the samples, where the state of deformation was approximately homogeneous, and the in-plane principal stretches *λ*_1_ and *λ*_2_ were computed. Synchronized acquisition of images and the data from a pressure sensor (digital manometer, LEX 1, Keller, Switzerland) for the EB test, or load cells (MTS Systems, Eden Prairie, USA) for UA tests, provided time histories of displacements, inflation pressure and forces^9, 26^. Nominal tension *T* in UA tests was obtained by dividing measured forces with sample width in the reference state. Test pieces for in situ characterization were cut to dimensions of 60 mm  × 10 mm (40 mm free length) for UA tests and to 55 mm diameter for inflation tests (free diameter 35 mm). Custom-made devices were used to perform in situ mechanical testing^54^. For UA experiments stretching was obtained using a motorized axis moving the clamps in opposite direction at a predefined speed to maintain the same sample region centered under the lens of a MPM (Fluoview 1000 MPE, Olympus, water objective: XLPlan N25×, NA 1.05). Applied strain (elongation) *ε* =  (*l*−*L*)/*L* was defined as the ratio between increase in free sample length, i.e., grip distance, and the length *L* in the reference state. Each experiment consisted of a series of loading steps of few seconds followed by an interval of several minutes to acquire 3D stacks with typical dimensions of 250 μm × 250 μm × 200 μm with an out-of-plane spacing of 3 μm, at one or multiple locations with an excitation wavelength of 820 nm. Image acquisition was started typically 60 s after each loading step. Previous experiments with hAM^9^ had shown that this interval was sufficient to reach stable sample dimensions. The acquisition time of a single image was about 4 s, so that the total time depended on the number of images with vertical spacing of 3 μm required to scan the whole thickness of the sample. Fluorescence of cell nuclei (stained with Hoechst 33342 or DAPI, Invitrogen) and the SHG signal of collagen were acquired using appropriate filters (Olympus FV10-MRROPT, BA397–412, BA455–490). Laser intensity, filter sensitivity and grayscale thresholds were adjusted in each application to optimize the contrast of the images. Microscopy images were processed with Imaris software (Bitplane AG, Zurich, Switzerland). Angular distribution histograms of collagen (Fig. 3c, d) were prepared with Matlab (TheMathWorks Inc., Natick, MA, USA), according to ref.^55^ from representative image stacks (cf. Fig. 1c, d). Monotonic EB tests were performed in volume control (Standard Infuse/Withdraw PHD Ultra Syringe Pump, Harvard Appartus, USA), cyclic tests under pressure control, achieved by setting the height of a water column loading the lower chamber of the inflation device (hAM) or by controlling the syringe pump with custom Lab View code (National Instruments, Huntsville, USA). The reference configuration was defined by a small tissue-specific reference force threshold (per width of the sample) of 0.33 N m^−1^ (hAM) or 6.7 N m^−1^ (bGC, pPC) in UA experiments, and by a small inflation pressure in the order 0.1 kPa for EB tests.

### Determination of volume change and Poisson’s ratios

Quantification of volume changes associated with in-plane loading of soft tissue membranes was achieved by combination of macroscopic (*λ*_1_ and *λ*_2_) and the in situ experimental data (*λ*_3_). Thickness measurements were based on the a sharp transition from high signal intensity to negligible detected emission at the upper and lower boundaries of 3D stacks in in situ tests^54^ (Fig. 1d). While the SHG signal intensity might change due to variation of optical properties over sample thickness, a sharp transition from significant to negligible SHG signal level was consistently observed and taken as a robust criterion to determine tissue thickness. The technique was further confirmed by UA experiments, in which bGC test pieces were rotated by 90° along their long axis such that the lens focused on planes perpendicular to the membrane plane, and stacks were acquired with a series of membrane cross sections in the vicinity of the edge (Fig. 1c). The ratio of deformed to reference thickness provided the thickness stretch *λ*_3_ which was combined with the in-plane stretches *λ*_1_ and *λ*_2_ to compute the volume change *J* = *λ*_1_ *λ*_2_ *λ*_3_ (Supplementary Fig. 1c, d). Apparent Poisson’s ratios were calculated as^13^

2 $${\nu _{12}} = - \frac{{{\lambda _1}}}{{{\lambda _2}}}\frac{{\partial {\lambda _2}}}{{\partial {\lambda _1}}},\,\,\,\,{\nu _{13}} = - \frac{{{\lambda _1}}}{{{\lambda _3}}}\frac{{\partial {\lambda _3}}}{{\partial {\lambda _1}}},$$

where the partial derivatives were approximated by the central difference quotient, or one-sided finite differences at the first and last data point. For the sake of comparability, Poisson’s ratios were evaluated at steps of 3% for both *λ*_1_−*λ*_3_ and *λ*_1_−*λ*_2_ data, despite the higher data density available for the latter (Supplementary Fig. 1e, f).

### Macroscopic stress relaxation tests with change of bath

On the basis of a protocol previously applied to bGC and hAM in physiological saline solution^9, 26^, UA test pieces were loaded rapidly ( ~ 1 s) up to a predefined force value (bGC: 0.8 N, hAM: 0.2 N, pPC: 0.8 N) and kept fixed at the corresponding displacement for 10 minutes. Top view images of the relaxing specimen and the continuously decreasing tension response were recorded. This standard protocol was modified such that after ~45 s of relaxation the bath was changed to distilled water (Fig. 2a–c). The hAM data were filtered with a moving average filter due to larger noise at lower forces.

### Confined compression tests with bGC

Seven to eight circular samples of 16 mm diameter were prepared from larger samples of bGC, stacked into an impermeable cylindrical cavity with 16 mm inner diameter, made of polymethylmethacrylate, and immersed in saline solution. A cylindrical porous glass filter (diameter 15 mm, P250 (ISO4793), Duran Group GmbH, Wertheim, Germany) was placed on top and the stack of membrane specimens was compressed in several steps using a vertical tension/compression testing device (Stentor II, Andilog Industries, Vitrolles, France), interrupted by hold phases to reach quasi-equilibrium (Fig. 2d). This arrangement corresponds to a confined compression test^30, 56^, in which the volume change *J* of the tissue is equal to the ratio between current and reference height of the tissue stack. The corresponding forces *F* were recorded and divided by the cross-section *A*_0_ of the stack to obtain the equilibrium stress. The initial steps led to zero equilibrium force, indicating that excessive liquid between the loosely piled membranes was expelled in this phase. The reference height was thus defined at the beginning of the step, for which we measured a nonzero force after a dwell time of 200 s.

### In situ tests on human amnion samples with defects

In situ inflation tests were performed on circular hAM specimens with a central circular hole of 1 mm (Fig. 4a), created by a biopsy puncher (Kai Medical, Seki, Japan). Defective amnion specimens were combined with the respective intact chorion layers to prevent leakage of water through the hole during inflation. The amnion-chorion double-layer was clamped such that the defect was positioned in the center of the inflated region. Inflation pressure was controlled based on the height of the corresponding water column. Images were acquired at the reference state (0.1 kPa) and at 6 following loading steps up to a pressure of about 5.5 kPa. Experiments with a crack-like defect in amnion were performed using specimens with free dimensions of 10 mm  × 40 mm, i.e., with length to width aspect ratio <1 and a lateral cut of 10 mm, based on the typical arrangement for mode-I fracture tests to determine the tearing energy^46, 57^ (Fig. 4a). The custom-built device for in situ tensile (UA) testing was used to progressively open the crack by displacing the clamps in steps of 0.5 mm at 0.1 mm s^−1^. The microscopy data were analyzed with Matlab (Version 2013a, TheMathWorks Inc., Natick, MA, USA). To measure the collagen signal intensity (CSI) in the near field of a defect, each image of the stack was converted to grayscale and the SHG intensity value in each pixel was added up along the *z*-coordinate. To identify the formation and dissolution of collagenous structures in the vicinity of a defect, the laser intensity and all image acquisition settings were kept constant over a series of images taken during loading and unloading of a notched specimen, and a normalized integrated CSI was computed by summing the CSI value in each pixel over the first 50 μm distance from the notch tip and normalizing by its maximum obtained during a load cycle (Fig. 4d, e). To compute the NAR^47, 58^ images were converted to binary, and the nuclei in a region of 0.1 mm^2^ were fitted with ellipses. NAR was then computed as the ratio between the major and minor axes of each ellipse, plotted against the distance from the defect site, and a moving average filter was applied to smooth the data. While this 2D analysis does not provide quantitative information on the change in nucleus shape, the determined spatial variation in NAR serves to quantify the size of the near field of a defect, based on the transition from elevated values at the defect site to a stable mean value in the far field. To compare NAR and CSI, the smoothed NAR curves were normalized by their maximum (Fig. 4b, c).

### 3D discrete fiber network model

A recent planar network model^59^ was extended to 3D. Representative volume elements (RVEs) were created by seeding a spatial domain of *V*_RVE_ = *b*_RVE_ × *b*_RVE_ × *t*_RVE_ randomly with *N*_I_ intersection points and connecting these, respectively, to other four intersections by straight lines. The amount of intersections *N*_I_ was controlled by defining the intersection density *ρ*_I_ so that

3 $${N_{\rm{I}}} = {\rho _{\rm{I}}}{V_{{\rm{RVE}}}} = {\rho _{\rm{I}}}(b_{{\rm{RVE}}}^2{t_{{\rm{RVE}}}}).$$

The connecting lines were generated using a weighted random selection process, where the weight *p* for each possible connector with length *l*, in-plane orientation *α* and out-of-plane orientation *ϕ* is given by a combination of probability distributions^60^

4 $$p = {l^{ - 2}}{p_{\rm{l}}}\left( l \right){p_{\rm{\alpha }}}\left( \alpha \right){p_\varphi }\left( \varphi \right)$$

with *p*_*α*_ = 1 to account for in-plane isotropy of the membranes, whereas *p*_*φ*_ was defined by a von-Mises distribution with concentration parameter *β*, and *p*_l_ is a Poisson distribution, leading to networks with Poisson distributed fiber lengths of average length *l*_s_. Fibers were discretized by connector elements (CONN3D2) in Abaqus/Standard software (Abaqus 6.10EF1, Dassault Systèmes Simulia Corp., Providence, RI, USA). To account for the resistance of the network against volumetric deformation a single solid continuum element (C3D8) was superimposed on the network, similar to ref. ^25^ but without any distortional stiffness. Its material properties were defined by the strain-energy function

5 $$\Phi \left( J \right) = \frac{\kappa }{2}\left( {1 - J^*} \right)\left( {\frac{{{{\left( {1 - J^*} \right)}^2}}}{{J - J^*}} + J + J^* - 2} \right),$$

where *κ* is a material parameter with dimension of stress. This model predicts increasing resistance to compression and limits the volume reduction to J*. Short range, soft elastic interaction between adjacent fibers through a weak matrix were represented by two additional connectors with low stiffness *k*_m_ between intersections, respectively, isotropically distributed but chosen as short as possible. The non linear response of the fiber connectors was approximated by a tri-linear force-strain (*F*_c_−*ε*_c_) curve (Fig. 3h), representing the low compressive stiffness (*k*_0_) for *ε*_c_<0, the moderate stiffness (*k*_b_) during the bending-dominated straightening phase (0<*ε*_c_<*ε*_s_), and the high tensile stiffness (*k*_1_) of straight fibers (*ε*_c_>*ε*_s_). The RVE was subjected to homogeneous boundary conditions reflecting the macroscopic displacement and stress-free lateral faces in UA and EB tests. The computed reaction forces on the boundaries were used to compute the homogenized stress tensor^61^ and the orientation of the connector elements in the deformed RVE were extracted, processed with Matlab (Version R2013a) and represented as polar plots.

The results in Fig. 3e–g, i–k are representative of bGC behavior with a thickness of 150 μm and reproduce the measured mean tension, elongation and contraction data in UA tests. The network was characterized by the following set of parameters: *ρ*_I_ = 0.00045 μm^−3^, *l*_s_ = 50 μm, *β* = 3, *k*_0_ = 9.6 μN, *k*_b_ = 19.2 μN, *k*_1_ = 32 mN, *k*_m_ = 16 μN, *ε*_s_ = 16.5%, *κ* = 0.06 MPa, J* = 0.2. Results in Fig. 3l for linear fiber behavior and isotropic fiber distributions were computed with *k*_b_ = *k*_0_ = *k*_1_ = 32 mN and *β* = 0, respectively, while maintaining the other parameters. To be consistent with the experimental procedures, a threshold stress of 0.044 MPa was used to identify the reference configuration, equivalent to the threshold force per width of 6.7 N m^−1^ used for experiments on bGC membranes.

### Biphasic chemo-mechanically coupled constitutive model

Combining theories for bi-phasic^62, 63^ and swelling^28, 64^ media, membranes were modeled as fluid-saturated biphasic tissues with reference solid and liquid volume fractions $\phi _{\rm{s}}^{{\rm{ref}}}$ and $\phi _{\rm{l}}^{{\rm{ref}}} = 1 - \phi _{\rm{s}}^{{\rm{ref}}}$, respectively (Supplementary Methods). For a history of deformation ***χ***(*X*,*t*) with deformation gradient ${\mathbf{F}} = {\rm{Grad}}_{\rm{\chi }}( {{\boldsymbol{X}},t} )$ at place ***X*** and local volume change *J* = det **F** of the mixture, the tissue Cauchy stress tensor was defined by^28, 30^

6 $${\mathbf{\sigma }} = {{\mathbf{\sigma }}_{\rm{s}}} - \left( {\pi + \bar \mu } \right){\mathbf{I}},$$

where **σ**_s_ is the stress contribution from the incompressible solid phase, *π* denotes the osmotic pressure, and $\bar \mu$ represents the chemical potential of water per current volume of the mixture. Following previous work^23, 26, 32^, the solid stress contribution **σ**_s_ is modeled as a fiber-reinforced, visco-hyperelastic material, quasi-isotropic in the membrane plane, such that

7 $${{\mathbf{\sigma }}_{\rm{s}}} = \phi _{\rm{s}}^{{\rm{ref}}}{c_0}{e^{q\left( {{g_{\rm{m}}} + {g_{{\rm{fe}}}}} \right)}}{J^{ - 1}}\left[ {{c_1}{\mathbf{F}}{{\mathbf{F}}^{\rm{T}}} + \frac{{{c_2}}}{N}\mathop {\sum}\limits_{i = 1}^N {{\left\langle {{\lambda _{{\rm{e}},i}} - 1} \right\rangle }^{2{c_3} - 1}}\frac{{{\lambda _{{\rm{e}},i}}}}{{|{\mathbf{F}}{{\boldsymbol{M}}_i}{|^2}}}{\mathbf{F}}{\boldsymbol{M}}_i} \otimes {\boldsymbol{F}}{\boldsymbol{M}}_i \right].$$

The unit vectors ***M***_*i*_ define the directions of *N* families of fibers in the reference state, uniformly distributed within the membrane plane and with an out-of-plane inclination of $\pm \vartheta$, respectively. *c*_0_, *c*_1_, *c*_2_, *c*_3_ and *q* are material parameters, $\langle \cdot \rangle$ denote Macaulay brackets, and

8 $${g_{\rm{m}}} = {c_1}\left[ {{\rm{tr}}\left( {{\mathbf{F}}{{\mathbf{F}}^{\rm{T}}}} \right) - 3} \right],\quad {g_{{\rm{fe}}}} = \frac{{{c_2}}}{{{c_3}}}\frac{1}{N}\mathop {\sum}\limits_{i = 1}^N {\left\langle {{\lambda _{{\rm{e}},i}} - 1} \right\rangle ^{2{c_3}}},$$

similar to ref. ^65^. The stretch in the *i*th fiber families is given by *λ*_e,*i*_ which calculate from evolution equations^32^

9 $$\frac{{{{\dot \lambda }_{{\rm{e}},i}}}}{{{\lambda _{{\rm{e}},i}}}} = \frac{{{{\dot \lambda }_i}}}{{{\lambda _i}}} - \phi _{\rm{s}}^{{\rm{ref}}}{c_0}{e^{q\left( {{g_{\rm{m}}} + {g_{{\rm{fe}}}}} \right)}}\frac{{{c_2}}}{{{\nu _{\rm{F}}}}}{\lambda _{{\rm{e}},i}}{\left\langle {{\lambda _{{\rm{e}},i}} - 1} \right\rangle ^{2{c_3} - 1}},\,\,\,\,\,i = 1,2,...,N,\,\,\,\,\,{\lambda _i} = \left| {{\mathbf{F}}{{\boldsymbol{M}}_i}} \right|$$

with initial conditions *λ*_e,*i*_ = 1 at *t* = 0 and *v*_F_ is a viscosity-like constant. The relationship between the osmotic pressure *π* and tissue volume change *J* was described by the empiric relation

10 $$\pi = \pi \left( J \right) = {a_0}{\left[ {\frac{{1 - \phi _{\rm{s}}^{{\rm{ref}}}}}{{J - \phi _{\rm{s}}^{{\rm{ref}}}}}} \right]^{2{a_1}}} + {\pi _0}$$

determined experimentally for bGC in confined compression tests, where *a*_0_, *a*_1_, and ${\pi _0} = \phi _{\rm{s}}^{{\rm{ref}}}{c_0}{c_1} - {a_0}$ are parameters. The liquid volume flux *q* follows from a generalized Darcy’s law with an isotropic but compaction-dependent conductivity^31^ and the gradient of^30^ $\bar \mu$ as

11 $${\boldsymbol{q}} = - {k_0}{\left[ {\frac{{J - \phi _{\rm{s}}^{{\rm{ref}}}}}{{1 - \varphi _{\rm{s}}^{{\rm{ref}}}}}} \right]^{{k_1}}}{e^{\frac{{{k_2}}}{2}\left( {{J^2} - 1} \right)}}{\kern 1pt} {\kern 1pt} {\rm{grad}}{\kern 1pt} \ \bar \mu ,$$

where *k*_0_ is the initial hydraulic conductivity in the reference state. The model was implemented in finite element software (COMSOL Multiphysics Version 5.2) and simulations were performed by specifying initial and boundary conditions. The model was calibrated by comparison with the relaxation test data on bGC samples (Fig. 2), and simulations were performed for *N* = 32 with the following parameter set: $\vartheta { = 10.36^ \circ }$, *q* = 5.0, *c*_0_ = 1.8 MPa, *c*_1_ = 0.001, *c*_2_ = 180, *c*_3_ = 1.02, *v*_F_ = 1.2121e5 MPa s, $\phi _{\rm{s}}^{{\rm{ref}}} = 0.15$, *a*_0_ = 0.9435 kPa, *a*_1_ = 1.0235, *k*_0_ = 3.4783 e−14 m^4^ N^−1^ s^−1^, *k*_1_ = 0.6, *k*_2_ = 9.8.

### Simulation of relaxation with change in bath salinity

A central 0.7 mm-long segment of the UA tension specimen with full width of 15 mm and thickness of 0.1 mm was simulated. Preliminary computations based on the entire specimen geometry showed that fluid motion along the stretching direction becomes negligible beyond this central region so that the computational domain could be reduced by preventing flux through the cutting surface. Due to the symmetry of the sample, one eighth of this segment was discretized with hexahedral elements, and zero flux and displacement boundary conditions were prescribed at the faces lying in symmetry planes, while on the boundaries in contact with the external bath zero stress and a defined value of the chemical potential was prescribed^30^ $\bar \mu = {\bar \mu _{{\rm{ext}}}}$. In line with the experimental protocol, the specimen was elongated by 5% within 0.5 s at constant rate and maintained at constant length for 600 s. To model the tissue response to a reduction of salinity in the surrounding bath, an increase of *π*(*J*) to *π*_ch_(*J*) was implemented at a prescribed time *t*_ch_ during the dwell phase, and parameters of *π*_ch_(*J*) were selected to approximate the corresponding experimental behavior after a change of bath (Fig. 2e).

### Equibiaxial loading of a membrane with circular defect

A square sample of tissue with side length 10 mm, thickness of 0.1 mm and a circular hole of 1 mm diameter was considered and an octant of this geometry was simulated by exploitation of symmetry (Fig.  4f). A defined value of the chemical potential and zero stress were prescribed on the surfaces in contact with the external bath, while the flux was prevented on the lateral surfaces, which were displaced by 8% of the side length within 4 s to create a state of EB extension in the membrane plane. This displacement was maintained and the deformation, stress, flux and pressure fields were analyzed over 30 s. The ratio of circumferential to radial stretch *λ*_*θ*_/*λ*_*r*_ with respect to a cylindrical coordinate system aligned with the cylindrical hole, and the local change in tissue density *J* ^−1^ were computed from the resultant deformation field. These quantities were normalized by their maximum values, respectively, so that for the density change *ρ*/max(*ρ*)  = min(*J*)/*J* (Fig. 4g).

### Data availability

The data that support the findings of this study are available from the corresponding authors on request.

## Electronic supplementary material

Supplementary Information

Description of Additional Supplementary Information

Supplementary Movie I

## References

[1] Vossoughi J, Vaishnav RN (1979). Comments on the paper “Volume compressibility of human abdominal skin”. J. Biomech..

[2] Chagnon G, Rebouah M, Favier D (2015). Hyperelastic energy densities for soft biological tissues: A review. J. Elast..

[3] Mow VC, Holmes MH, Michael Lai WM (1984). Fluid transport and mechanical properties of articular cartilage: A review. J. Biomech..

[4] Biot MA (1962). Mechanics of deformation and acoustic propagation in porous media. J. Appl. Phys..

[5] Baughman RH, Stafstrom S, Cui C, Dantas SO (1998). Materials with negative compressibilities in one or more dimensions. Science.

[6] Lanir Y, Salant EL, Foux A (1988). Physico-chemical and microstructural changes in collagen fiber bundles following stretch in-vitro. Biorheology.

[7] Hannafin JA, Arnoczky SP (1994). Effect of cyclic and static tensile loading on water content and solute diffusion in canine flexor tendons: an in vitro study. J. Orthop. Res..

[8] Reese SP, Weiss JA (2013). Tendon fascicles exhibit a linear correlation between Poisson’s ratio and force during uniaxial stress relaxation. J. Biomech. Eng..

[9] Mauri A, Perrini M, Ehret AE, De Focatiis DS, Mazza E (2015). Time-dependent mechanical behavior of human amnion: Macroscopic and microscopic characterization. Acta Biomater..

[10] Perrini M (2015). Mechanical and microstructural investigation of the cyclic behavior of human amnion. J. Biomech. Eng..

[11] Woo SL-Y (1979). Large deformation nonhomogeneous and directional properties of articular cartilage in uniaxial tension. J. Biomech..

[12] Brown AEX, Litvinov RI, Discher DE, Purohit PK, Weisel J (2009). Multiscale mechanics of fibrin polymer: Gel stretching with protein unfolding and loss of water. Science.

[13] Lake SP, Barocas VH (2011). Mechanical and structural contribution of non-fibrillar matrix in uniaxial tension: A collagen-agarose co-gel model. Ann. Biomed. Eng..

[14] Lai VK, Lake SP, Frey CR, Tranquillo RT, Barocas VH (2012). Mechanical behavior of collagen-fibrin co-gels reflects transition from series to parallel interactions with increasing collagen content. J. Biomech. Eng..

[15] Roeder BA, Kokini K, Robinson JP, Voytik-Harbin SL (2004). Local, three-dimensional strain measurements within largely deformed extracellular matrix constructs. J. Biomech. Eng..

[16] Adeeb S, Ali A, Shrive N, Frank C, Smith D (2004). Modelling the behaviour of ligaments: A technical note. Comput. Methods Biomech. Biomed. Engin..

[17] Lanir Y (2012). Osmotic swelling and residual stress in cardiovascular tissues. J. Biomech..

[18] Masic A (2015). Osmotic pressure induced tensile forces in tendon collagen. Nat. Commun..

[19] Meinert M (2001). Proteoglycans and hyaluronan in human fetal membranes. Am. J. Obstet. Gynecol..

[20] Stingl J (2002). Morphology and some biomechanical properties of human liver and spleen. Surg. Radiol. Anat..

[21] Naimark WA, Lee JM, Limeback H, Cheung DT (1992). Correlation of structure and viscoelastic properties in the pericardia of four mammalian species. Am. J. Physiol..

[22] Hopf R (2016). Experimental and theoretical analyses of the age-dependent large-strain behavior of Sylgard 184 (10:1) silicone elastomer. J. Mech. Behav. Biomed. Mater..

[23] Bürzle W, Mazza E (2013). On the deformation behavior of human amnion. J. Biomech..

[24] Purohit PK, Litvinov RI, Brown AEX, Discher DE, Weisel J (2011). Protein unfolding accounts for the unusual mechanical behavior of fibrin networks. Acta Biomater..

[25] Lake SP, Hadi MF, Lai VK, Barocas VH (2012). Mechanics of a fiber network within a non-fibrillar matrix: Model and comparison with collagen-agarose co-gels. Ann. Biomed. Eng..

[26] Bircher K, Ehret AE, Mazza E (2016). Mechanical characteristics of bovine Glisson’s capsule as a model tissue for soft collagenous membranes. J. Biomech. Eng..

[27] Kovach IS (1995). The importance of polysaccharide configurational entropy in determining the osmotic swelling pressure of concentrated proteoglycan solution and the bulk compressive modulus of articular cartilage. Biophys. Chem..

[28] Lanir Y (1987). Biorheology and fluid flux in swelling tissues. I. Bicomponent theory for small deformations, including concentration effects. Biorheology.

[29] Ateshian GA, Rajan V, Chahine NO, Canal CE, Hung CT (2009). Modeling the matrix of articular cartilage using a continuous fiber angular distribution predicts many observed phenomena. J. Biomech. Eng..

[30] Wilson W, Van Donkelaar CC, Huyghe JM (2005). A comparison between mechano-electrochemical and biphasic swelling theories for soft hydrated tissues. J. Biomech. Eng..

[31] Holmes MH, Mow VC (1990). The nonlinear characteristics of soft gels and hydrated connective tissues in ultrafiltration. J. Biomech..

[32] Mauri A, Ehret AE, De Focatiis DSA, Mazza E (2016). A model for the compressible, viscoelastic behavior of human amnion addressing tissue variability through a single parameter. Biomech. Model. Mechanobiol..

[33] Swartz MA, Fleury ME (2007). Interstitial flow and its effects in soft tissues. Annu. Rev. Biomed. Eng..

[34] Donnan FG (1924). The theory of membrane equilibria. Chem. Rev..

[35] McGrail DJ (2015). Osmotic regulation is required for cancer cell survival under solid stress. Biophys. J..

[36] Stewart MP (2011). Hydrostatic pressure and the actomyosin cortex drive mitotic cell rounding. Nature.

[37] Danziger J, Zeidel ML (2015). Osmotic homeostasis. Clin. J. Am. Soc. Nephrol..

[38] Kabla A, Mahadevan L (2007). Nonlinear mechanics of soft fibrous networks. J. R. Soc. Interface.

[39] Janmey PA (2007). Negative normal stress in semiflexible biopolymer gels. Nat. Mater..

[40] Yang W (2015). On the tear resistance of skin. Nat. Commun..

[41] Taylor D, O’Mara N, Ryan E, Takaza M, Simms C (2012). The fracture toughness of soft tissues. J. Mech. Behav. Biomed. Mater..

[42] Sen D, Buehler MJ (2011). Structural hierarchies define toughness and defect-tolerance despite simple and mechanically inferior brittle building blocks. Sci. Rep.

[43] Koh CT, Oyen ML (2012). Branching toughens fibrous networks. J. Mech. Behav. Biomed. Mater..

[44] Mai Y-W, Atkins AG (1989). Further comments on J-shaped stress-strain curves and the crack resistance of biological materials. J. Phys. D: Appl. Phys.

[45] Devlieger R, Millar LK, Bryant-Greenwood G, Lewi L, Deprest JA (2006). Fetal membrane healing after spontaneous and iatrogenic membrane rupture: a review of current evidence. Am. J. Obstet. Gynecol..

[46] Rivlin RS, Thomas AG (1953). Rupture of rubber. I. Characteristic energy for tearing. J. Polym. Sci..

[47] Huang HY, Liao J, Sacks MS (2007). In-situ deformation of the aortic valve interstitial cell nucleus under diastolic loading. J. Biomech. Eng..

[48] Versaevel M, Grevesse T, Gabriele S (2012). Spatial coordination between cell and nuclear shape within micropatterned endothelial cells. Nat. Commun..

[49] Lutolf MP, Hubbell JA (2005). Synthetic biomaterials as instructive extracellular microenvironments for morphogenesis in tissue engineering. Nat. Biotechnol..

[50] Noselli G, Lucantonio A, McMeeking RM, Desimone A (2016). Poroelastic toughening in polymer gels: A theoretical and numerical study. J. Mech. Phys. Solids..

[51] Humphrey J, Dufresne ER, Schwartz MA (2014). Mechanotransduction and extracellular matrix homeostasis. Nat. Rev. Mol. Cell. Biol..

[52] Casares L (2015). Hydraulic fracture during epithelial stretching. Nat. Mater..

[53] Mazza E, Ehret AE (2015). Mechanical biocompatibility of highly deformable biomedical materials. J. Mech. Behav. Biomed. Mater..

[54] Mauri A (2015). Deformation mechanisms of human amnion: Quantitative studies based on second harmonic generation microscopy. J. Biomech..

[55] Feng XG, Milanfar P (2002). Multiscale principal components analysis for image local orientation estimation. Conf. Rec. Asilomar Conf. Signals Syst. Comput..

[56] Soltz MA, Ateshian GA (1998). Experimental verification and theoretical prediction of cartilage interstitial fluid pressurization at an impermeable contact interface in confined compression. J. Biomech..

[57] Sun JY (2012). Highly stretchable and tough hydrogels. Nature.

[58] Nathan AS, Baker BM, Nerurkar NL, Mauck RL (2011). Mechano-topographic modulation of stem cell nuclear shape on nanofibrous scaffolds. Acta Biomater..

[59] Mauri A, Hopf R, Ehret AE, Picu CR, Mazza E (2016). A discrete network model to represent the deformation behavior of human amnion. J. Mech. Behav. Biomed. Mater..

[60] Sampson, W. W. *Modelling Stochastic Fibrous Materials with Mathematica* (Springer, 2009).

[61] Stylianopoulos T, Barocas VH (2007). Volume-averaging theory for the study of the mechanics of collagen networks. Comput. Meth. Appl. Mech. Eng.

[62] de Boer R (2000). Contemporary progress in porous media theory. ASME Appl. Mech. Rev..

[63] Federico S, Grillo A (2012). Elasticity and permeability of porous fibre-reinforced materials under large deformations. Mech. Mater..

[64] Hong W, Liu Z, Suo Z (2009). Inhomogeneous swelling of a gel in equilibrium with a solvent and mechanical load. Int. J. Solids Struct..

[65] Rubin MB, Bodner SR (2002). A three-dimensional nonlinear model for dissipative response of soft tissue. Int. J. Solids Struct..

