# Supplementary material for: Inverse poroelasticity as a fundamental mechanism in biomechanics and mechanobiology
Source: Nat Commun. 2017 Oct 17;8:1002. doi: 10.1038/s41467-017-00801-3 (PMC5714996; doi:10.1038/s41467-017-00801-3)
Supplement: Supplementary file 1 — Supplementary Information [file 41467_2017_801_MOESM1_ESM.pdf]

Supplementary Information

Supplementary Methods

Formulation of the bi-phasic chemo-mechanically coupled constitutive model

This section describes the formulation of the bi-phasic model presented in Eqs. 6-11 in the Methods. The reference state of the membranes is characterized by the solid and liquid volume fractions $\phi_{\mathrm{s}}^{\mathrm{ref}}$ and $\phi_{\mathrm{l}}^{\mathrm{ref}} = 1 - \phi_{\mathrm{s}}^{\mathrm{ref}}$, respectively[1–3]. For a history of deformation given by the deformation gradient $\mathbf{F} = \mathbf{F}(\boldsymbol{X}, t)$, the local volume change of the mixture is given by $J = \det \mathbf{F}$ and the solid and liquid volume fractions change to their current values $\phi_{\mathrm{s}} = J^{-1}\phi_{\mathrm{s}}^{\mathrm{ref}}$ and $\phi_{\mathrm{l}} = 1 - J^{-1}\phi_{\mathrm{s}}^{\mathrm{ref}}$.
The Cauchy stress tensor of such a bi-phasic material is given by[1–3]

$$\boldsymbol{\sigma} = \boldsymbol{\sigma}_{\mathrm{s}} - p\mathbf{I}, \tag{1}$$

where $\boldsymbol{\sigma}_{\mathrm{s}}$ is the stress contribution from the incompressible solid phase and p is the pore pressure acting on the incompressible inviscid fluid phase. We combined this theory with the modeling of swelling media, for which a Helmholtz free energy density per unit reference volume of the material can be defined as[4]

$$\Psi = \check{\Psi}(\mathbf{F}, C), \tag{2}$$

where C is the molar concentration of solvent per unit reference volume. A Legendre transform leads to a representation of the free energy in terms of $\mathbf{F}$ and the chemical potential μ of the solvent[4,5]

$$\hat{\Psi}(\mathbf{F}, \mu) = \check{\Psi}(\mathbf{F}, C(\mathbf{F}, \mu)) - \mu C, \quad \mu = \frac{\partial \check{\Psi}(\mathbf{F}, C)}{\partial C}. \tag{3}$$

For a porous saturated bi-phasic medium, the molar solvent concentration C in Supplementary Eq. (2) is related to the ratio between current volume of liquid and reference volume of the mixture by the liquid molar volume V_{m} such that

$$C = \frac{J}{V_{\mathrm{m}}}\phi_{\mathrm{l}} = \frac{J}{V_{\mathrm{m}}}(1 - \phi_{\mathrm{s}}) = \frac{J}{V_{\mathrm{m}}}(1 - J^{-1}\phi_{\mathrm{s}}^{\mathrm{ref}}) = \frac{1}{V_{\mathrm{m}}}(J - \phi_{\mathrm{s}}^{\mathrm{ref}}). \tag{4}$$

Our previous results indicated that the solid phase of the tissues is not elastic but creeps upon constant load[6], and, accordingly, a viscoelastic model was considered to describe the behavior of collagen fibers[7], which uses a set $\{\alpha\}$ of internal variables[8] to account for the viscoelastic behavior. With this and combining Supplementary Eqs. (3) and (4), the free energy density of the mixture reads

$$\hat{\Psi}(\mathbf{F}, \{\alpha\}, \mu) = \hat{\Psi}_{\mathrm{s}}(\mathbf{F}, \{\alpha\}) + \hat{\Psi}_{\mathrm{x}}(J, \mu) - \frac{\mu}{V_{\mathrm{m}}}(J - \phi_{\mathrm{s}}^{\mathrm{ref}}). \tag{5}$$

The first part accounts for the energy stored by deformation of the solid skeleton of the tissue. The second energy contribution relates to chemo-mechanical interactions between solute and solvent, and the third one results from the Legendre transformation described above. It is noteworthy that Supplementary Eq. (5) and all its contributions are defined per unit reference volume of the mixture in a

physiological saline bath and free of external mechanical loads. This state is preferred as a reference over the dry state ($\phi_s = 1$), which is hypothetical for soft tissues. From Supplementary Eq. (5), the corresponding Cauchy stress tensor $\boldsymbol{\sigma}$ calculates as

$$\boldsymbol{\sigma} = \boldsymbol{\sigma}_s - (\pi + \bar{\mu})\,\mathbf{I}, \quad \pi = -\frac{\partial \hat{\Psi}_x(J,\mu)}{\partial J}, \quad \bar{\mu} = \frac{\mu}{V_m}, \tag{6}$$

which defines p in Supplementary Eq. (1) by $p = \pi + \bar{\mu}$, and $\bar{\mu}$ represents the chemical potential of water per current volume of the mixture[9].

Following our previous work[7,10,11], the solid stress contribution $\boldsymbol{\sigma}_s$ is modeled as a fiber-reinforced, visco-hyperelastic material where the fibers are represented by N representative families. Their directions in the reference state are given by unit vectors $\boldsymbol{M}_i$, uniformly distributed within the membrane plane in an equiangular manner and with an out-of-plane inclination of $\pm\vartheta$, respectively. This arrangement leads to quasi-isotropy in the membrane plane and isotropy is approached for increasing values of[7] N. To take into account creep of fibres[6], the actual elastic stretches $\lambda_{e,i}$ of the fiber families, that lead to elastically stored energy, were considered as internal variables $\{\alpha\} = \{\lambda_{e,1}, \lambda_{e,2}, ..., \lambda_{e,N}\}$ and determined from evolution equations. The free energy function $\hat{\Psi}_s$ of the solid in Supplementary Eq. (5) is thus given by

$$\hat{\Psi}_s(\mathbf{F}, \lambda_{e,1}, \lambda_{e,2}, ..., \lambda_{e,N}) = \phi_s^{ref} \frac{c_0}{2q}\left[e^{q(g_m + g_{fe})} - 1\right], \tag{7}$$

based on the theory proposed in[12], and is specified by the two functions (Eq. 8)

$$g_m = c_1\left[\mathrm{tr}(\mathbf{F}\mathbf{F}^T) - 3\right], \quad g_{fe} = \frac{c_2}{c_3}\frac{1}{N}\sum_{i=1}^{N}\langle\lambda_{e,i} - 1\rangle^{2c_3}, \tag{8}$$

similar to ref[7,10], where c_0, c_1, c_2, c_3 and q are material parameters and $\langle\cdot\rangle$ denote Macaulay brackets. Exploiting the dissipation principle, the solid Cauchy stress tensor $\boldsymbol{\sigma}_s$ (Eq. 7) is obtained. The thermodynamically consistent evolution equations for the elastic fiber stretches $\lambda_{e,i}$ (Eq. 9) represent a special case discussed in ref[7].

Instead of the energy term $\hat{\Psi}_x$ in Supplementary Eq. (5), models for hydrated soft tissues rather define the osmotic contribution π in Supplementary Eq. (6), accounting for charge-dependent Donnan and charge-independent entropic osmotic effects that are caused by the presence of negatively charged proteoglycans within the solid tissue matrix[13–15]. In the present work, the relationship between the osmotic contribution $\pi(J)$ of the model and tissue volume change was determined experimentally for bGC in confined compression tests. Since there is no contribution of the collagen network in this situation of confined compression due to the Macaulay brackets in the formulation of Supplementary Eq. (8), the equilibrium model response is governed solely by the non-fibrous part in (Eq. 7) and the osmotic contribution π. We captured the latter by the empiric relation (Eq. 10)

$$\pi(J) = a_0\left[\frac{1 - \phi_s^{ref}}{J - \phi_s^{ref}}\right]^{2a_1} + \pi_0 \tag{9}$$

with parameters a_0, a_1, and $\pi_0 = \phi_s^{ref} c_0 c_1 - a_0$ guarantees that the overall stress in Supplementary Eq. (6) is zero in the reference state at equilibrium. Finally, the flow of water through the porous tissue is

described by a generalized Darcy's law. The liquid volume flux $\boldsymbol{q}$ is determined by a conductivity tensor $\mathbf{k}$ and the gradient of the difference between hydrostatic and osmotic pressure contributions, so that[9]

$$\boldsymbol{q} = -\mathbf{k}\,\mathrm{grad}\,(p - \pi) = -\mathbf{k}\,\mathrm{grad}\bar{\mu}. \tag{10}$$

When the tissue is compacted, the space between the collagen network decreases, which leads to a reduction of the permeability for the interstitial fluid. This is reflected by a dependence of the hydraulic conductivity on tissue volume change as[16]

$$\mathbf{k} = k_0 \left[\frac{J - \phi_{\mathrm{s}}^{\mathrm{ref}}}{1 - \phi_{\mathrm{s}}^{\mathrm{ref}}}\right]^{k_1} e^{\frac{k_2}{2}(J^2-1)}\mathbf{I}, \tag{11}$$

where k_0 is the initial hydraulic conductivity in the reference state. The model described in Supplementary Eqs. (1-11) was implemented in finite element software (COMSOL® Multiphysics Version 5.2). The unknowns of the problem are the deformation field $\boldsymbol{x} = \chi(\boldsymbol{X}, t)$ and the chemical potential $\bar{\mu}(\boldsymbol{X}, t)$. The set of equations to be solved is given by the balance of the linear momentum of the mixture and the mass balance for the liquid phase, which couples the deformation rate with the fluid motion through the Supplementary Eq. (10). The system of equations reads, cf. ref[5],

$$\mathrm{div}\boldsymbol{\sigma} = 0, \quad \dot{J} = J\mathrm{div}(\mathbf{k}\mathrm{grad}\bar{\mu}) \tag{12}$$

and the problem definition is completed by specifying the initial and boundary conditions. The model was calibrated by comparison with relaxation test data on bGC samples (Fig. 2b,d).

Simulation of hydraulic loading on superficial cell layers

The model was employed to investigate the consequences of the inverse poroelastic effect on superficial cell layers. In a recent work, Casares et al.[5] report on fracture of epithelial cell layers, cultured on top of PAA hydrogels, caused by the hydraulic loads during unloading of the substrate from a stretched equilibrium state. To illustrate that the inverse poroelastic behavior displayed by soft tissue membranes will, conversely, lead to these hydraulic loads during stretching, and not release, of the substrate, simulations were performed using a simplified version of the bi-phasic model described in Supplementary Eqs. (1-11), in which the effective behavior of the contribution of $\pi(J)$ was lumped in the strain-energy term g_{m} such that

$$g_{\mathrm{m}} = c_1 \left[\mathrm{tr}(\mathbf{F}\mathbf{F}^{\mathrm{T}}) - 3 - \frac{1}{c_5}(J^{-2c_5} - 1)\right], \tag{13}$$

according to a compressible neo-Hookean model and a penalization term[3] was included to guarantee that $\Psi \to \infty$ as $J \to \phi_{\mathrm{s}}^{\mathrm{ref}}$, see ref[17] for details. This formulation allowed in a simple way to choose between inverse poroelastic behavior and classical poroelasticity, as displayed by PAA, by considering ($c_2 \neq 0$) or neglecting the fiber contribution ($c_2 = 0$) in Supplementary Eq. (7), respectively. Following the benchmark problem by Casares et al.[5], a cylindrical domain of a membrane with height H=0.156 mm and radius R=0.1 mm was considered. Making use of the axisymmetry of the problem, a rectangular cross-section was modeled and discretized by triangular elements in COMSOL (Supplementary Fig.

2a). The cylinder was extended radially by 10% within 2 s at constant rate by prescribing the radial displacement of the outer faces, emulating an equibiaxial stretching load. A defined central region on the top face of the domain was considered impermeable, in order to simulate the presence of an island of epithelial cells (r_e=0.04mm, Supplementary Fig. 2a), and furthermore zero flux was assumed through the bottom and the lateral faces[5]. The remaining part of the top boundary ($r_e < r < R$) is permeable with prescribed chemical potential $\mu = \mu_{ext} = 0$. A load-unload cycle with intermediate and terminal hold phases of 600 s, respectively, was simulated (Supplementary Fig. 2c), the pressure and flux fields were analyzed (Supplementary Fig. 2b) and the pressure at the bottom of the cell cluster was evaluated (Supplementary Fig. 2d,e). The model for PAA-like behavior was parametrized such that pressures were obtained in a range observed in the experiments by Casares et al.[5] (Supplementary Fig. 2d).

Supplementary Discussion

Lateral contraction and volume reduction of soft tissues in tensile load states

The decreasing volume occurring in a uniaxial tension experiment, in which the hydrostatic pressure is negative, raises questions of thermodynamic consistency. As a limit case of our modeling assumption, fibers may be considered bi-linear with zero compressive and high tensile stiffness. For a network with isotropic distribution of straight fibers, a small strain uniaxial tensile experiment would lead to Poisson's ratio $\nu > 0.5$ and hence an apparently negative compression modulus, violating the thermodynamic restrictions for an isotropic linear elastic material. However, such conclusions are erroneous since the material response cannot be linearized at the reference state due to the discontinuity in stiffness. For some crystal structures and fluid-filled porous materials, on the other hand, an overall 'negative compressibility' of the mixture is known to comply with thermodynamic principles[18,19]. In the non-linear regime, hyperelasticity captures these effects in agreement with thermodynamics. Effectively, the large difference between compressive and tensile fiber stiffness leads to a pronounced non-linearity of the material behavior on a tissue scale. Hence, consider the simple example of an isotropic hyperelastic material with strain-energy function

$$\Psi = W(I_1) + U(J) \tag{14}$$

with

$$W(I_1) = \frac{c_1}{c_2}\left[e^{c_2(I_1-3)} - 1\right], \quad U(J) = -2c_1 \ln J, \tag{15}$$

where $I_1 = \lambda_1^2 + \lambda_2^2 + \lambda_3^2$, $J = \lambda_1\lambda_2\lambda_3$. This is a typical strain-stiffening material[20,21] which contains the compressible neo-Hookean model[22] as limit case if $c_2 \to 0$. Due to the convexity of $W(I_1)$ and $U(J)$, and monotonicity of $W(I_1)$ such a model can be shown to yield a materially stable response[23,24]. For uniaxial tension $(\lambda_1 \geq 1)$ isotropy suggests $\lambda_2 = \lambda_3$ and the principal Cauchy stresses read

$$\sigma_i = 2J^{-1}\lambda_i^2 \frac{\partial W(I_1)}{\partial I_1} + U'(J) = 2c_1 J^{-1}\left(\lambda_i^2 e^{c_2\left(\lambda_1^2 + 2J/\lambda_1 - 3\right)} - 1\right), \quad i = 1, 2, 3. \tag{16}$$

The boundary conditions of traction free lateral faces $(\sigma_2 = \sigma_3 = 0)$ require that the term in parentheses vanishes for $i = 2, 3$, while the tensile stress is $\sigma_1 = \sigma$. Whether this leads to an increase $(J > 1)$ or decrease $(J < 1)$ in volume for a given tensile stretch λ_1 depends on the parameter c_2, that governs the nonlinearity of the response. In fact, volume decrease occurs for higher material non-linearity, while the hydrostatic pressure $-\sigma/3$ is throughout negative (Supplementary Fig. 3a,b). For soft tissues in a static equilibrium, the osmotic pressure has a similar shape as $-U'(J)$, independent of its particular physical origin (charge-dependent or independent). Its contribution is low and only weakly sensible to volume changes in the dilute regime, while it increases rapidly when the liquid volume fraction becomes small. Since the liquid volume fraction is high in the reference state of soft tissues, already moderate changes in hydrostatic pressure require significant changes in J.

Noteworthy, the characteristic of high tensile vs. low compressive stiffness of fibers in a network was also made responsible for the negative Poynting effect in biopolymer gels[25], causing tensile stresses

normal to the sheared faces in simple shear experiments. On a continuum scale, it was shown that the apparently unusual behavior of these gels could be explained by the classical theory of hyperelasticity when giving up some additional empirical restrictions on the constitutive equations that are typically valid for rubber-like materials[26]. Similarly, we have shown here that volume reduction upon tensile loading is a characteristic that seems counterintuitive but is admissible within the classical theory of hyperelasticity.

Supplementary Figures

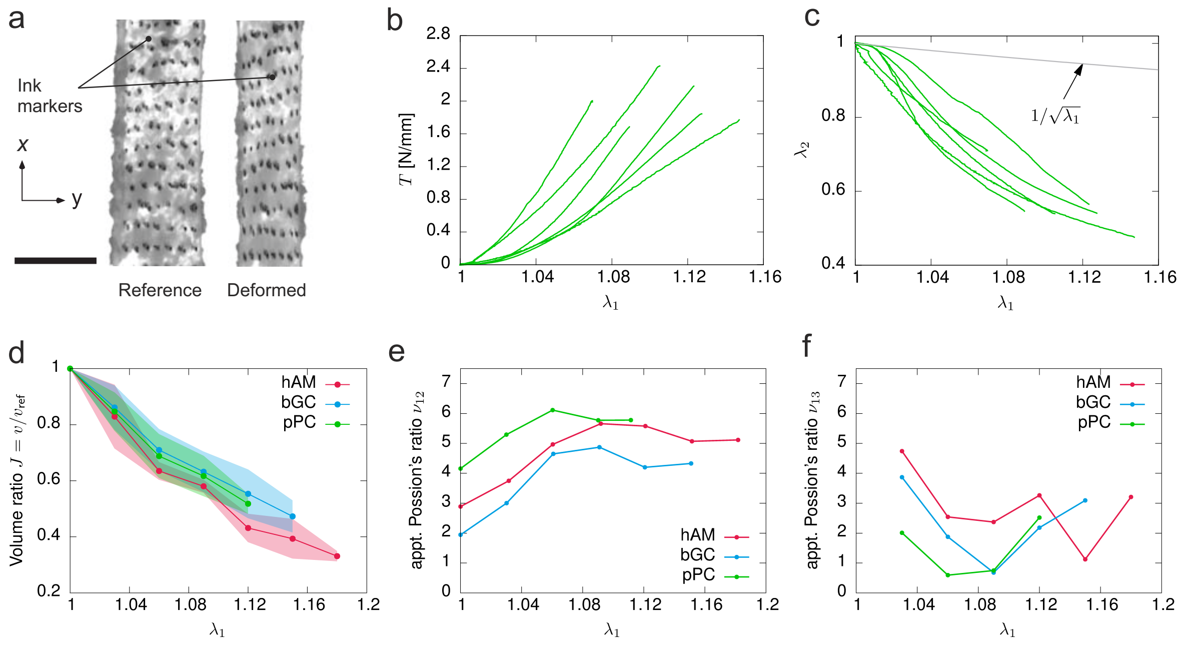

Supplementary Fig. 1 Mechanical behavior and volume change with tensile loading. **a** Strong lateral contraction is shown by top-view images of a representative test on bovine Glisson's capsule (bGC) in the reference and deformed ($\lambda_1 = 1.1$) state. The black markers are used for local stretch analysis. *Scale bar*: 10 mm. **b** Nominal tension vs. local stretch curves in monotonic uniaxial (UA) experiments for porcine pericardium (pPC, $n = 6$), showing similar characteristics as human amnion (hAM) [10,27] and bGC [11]. The local stretches λ_1 and λ_2 are obtained from the displacements of a set of ink markers on the sample surface by means of a custom written optical flow tracker algorithm [28] (**a**). **c** In-plane kinematic response λ_1 vs. λ_2 of the tests in (**b**); the expected lateral stretch $\lambda_2 = 1/\sqrt{\lambda_1}$ for an isotropic incompressible material is indicated for comparison. **d** The volume ratio J, obtained by combining macroscopic data (**c**) with the thickness stretch (λ_3) from multiphoton microscope (MPM) UA experiments, is represented by the mean and standard deviation with shaded error bars. Using data from refs [10,11,27] for hAM and bGC, and combining the latter with MPM measurements to retrieve thickness information highlight the similarity of these tissues in terms of the substantial volume change that they experience upon uniaxial extension. **e,f** Apparent in-plane ν_{12} and out-of-plane ν_{13} Poisson's ratios calculated from mean λ_2 vs. λ_1 and λ_3 vs. λ_1 data, respectively, for all three membranes.

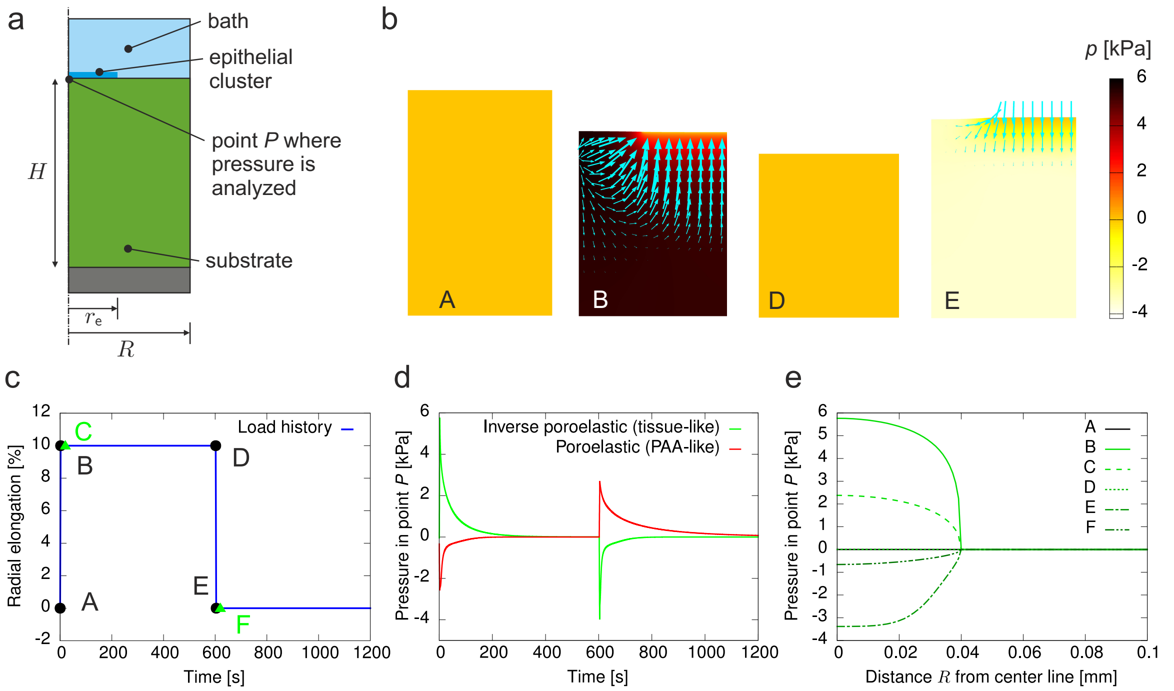

Supplementary Fig. 2 Simulation of hydraulic loading on epithelial cell layer. **a** Problem setup for simulation (cf. ref[5]): an axisymmetric substrate domain of height H and radius R with an epithelial cell cluster of radius r_e was rapidly stretched radially (2s) held at constant stretch for 600s, unloaded (2s) and kept in the unloaded state (600s). **b** Pressure distribution (*color bar*) and fluid flux (*arrows*) at the beginning and end of the loading (A to B) and unloading (D to E) phase respectively. At the permeable faces, water moves outward during loading and hold, and inward during and after unloading. **c** Applied load history in terms of applied radial elongation; letters indicate the time points at which flux and pressure are displayed in (**b**) and (**e**). **d** Fluid pressure at the center of the interface between substrate and epithelial layer. Pressure increase is observed during loading for tissue-like, inverse poroelastic substrates, opposite to PAA-like materials. **e** Pressure profiles over distance from center line at the time points indicated in (**c**).

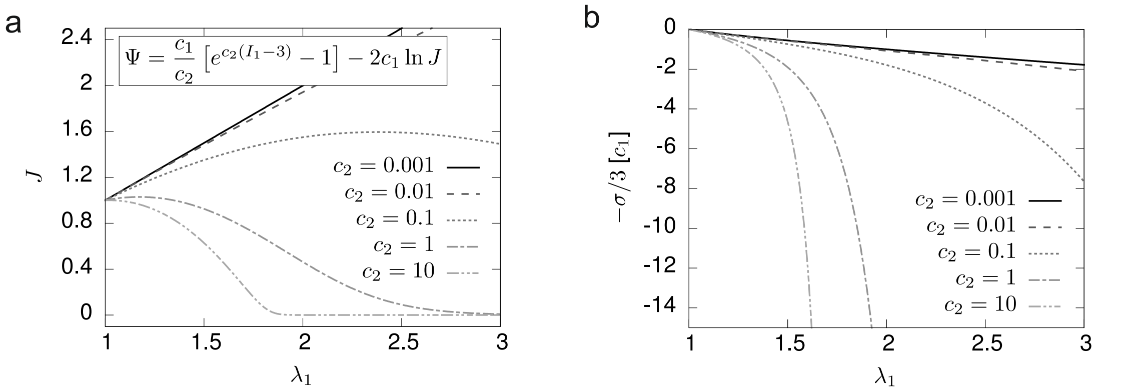

Supplementary Fig. 3 Negative compressibility of isotropic hyperelastic materials. **a,b** Volume change (**a**) and corresponding hydrostatic pressure (**b**) for an isotropic compressible hyperelastic material with variation of the parameter c_2 in the strain energy function Ψ (*inset*, Supplementary Eqs. 14, 15) illustrates that negative hydrostatic pressure can accompany both volume increase and decrease.

Supplementary References

1. de Boer, R. Contemporary progress in porous media theory. *ASME Appl. Mech. Rev.* **53**, 323–370 (2000).

2. Ehlers, W. Foundations of multiphasic and porous materials. In *Porous Media: Theory, Experiments and Numerical Applications*, (edited by Ehlers, W. & Bluhm, J.), pp. 3–86. Springer, Berlin, Heidelberg (2002).

3. Federico, S. & Grillo, A. Elasticity and permeability of porous fibre-reinforced materials under large deformations. *Mech. Mater.* **44**, 58–71 (2012).

4. Hong, W., Liu, Z. & Suo, Z. Inhomogeneous swelling of a gel in equilibrium with a solvent and mechanical load. *Int. J. Solids Struct.* **46**, 3282–3289 (2009).

5. Casares, L. et al. Hydraulic fracture during epithelial stretching. *Nat. Mater.* **14**, 343–351 (2015).

6. Mauri, A., Perrini, M., Ehret, A. E., De Focatiis, D. S. A. & Mazza, E. Time-dependent mechanical behavior of human amnion: Macroscopic and microscopic characterization. *Acta Biomater.* **11**, 314–323 (2015).

7. Mauri, A., Ehret, A. E., De Focatiis, D. S. A. & Mazza, E. A model for the compressible, viscoelastic behavior of human amnion addressing tissue variability through a single parameter. *Biomech. Model. Mechanobiol.* **15**, 1005–1017 (2016).

8. Coleman, B. D. & Gurtin, M. E. Thermodynamics with internal state variables. *J. Chem. Phys.* **47**, 597–613 (1967).

9. Wilson, W., Van Donkelaar, C. C. & Huyghe, J. M. A comparison between mechano-electrochemical and biphasic swelling theories for soft hydrated tissues. *J. Biomech. Eng.* **127**, 158–165 (2005).

10. Bürzle, W. & Mazza, E. On the deformation behavior of human amnion. *J. Biomech.* **46**, 1777–1783 (2013).

11. Bircher, K., Ehret, A. E. & Mazza, E. Mechanical characteristics of bovine Glisson's capsule as a model tissue for soft collagenous membranes. *J. Biomech. Eng.* **138**, 081005 (2016).

12. Rubin, M. B. & Bodner, S. R. A three-dimensional nonlinear model for dissipative response of soft tissue. *Int. J. Solids Struct.* **39**, 5081–5099 (2002).

13. Lanir, Y. Biorheology and fluid flux in swelling tissues. I. Bicomponent theory for small deformations, including concentration effects. *Biorheology* **24**, 173–187 (1987).

14. Kovach, I. S. The importance of polysaccharide configurational entropy in determining the osmotic swelling pressure of concentrated proteoglycan solution and the bulk compressive modulus of articular cartilage. *Biophys. Chem.* **53**, 181–187 (1995).

15. Ateshian, G. A., Rajan, V., Chahine, N. O., Canal, C. E. & Hung, C. T. Modeling the matrix of articular cartilage using a continuous fiber angular distribution predicts many observed phenomena. *J. Biomech. Eng.* **131**, 061003 (2009).

16. Holmes, M. H. & Mow, V. C. The nonlinear characteristics of soft gels and hydrated connective tissues in ultrafiltration. *J. Biomech.* **23**, 1145–1156 (1990).

17. Stracuzzi, A. & Ehret, A. E. Bi-phasic theory vs. volumetric viscoelasticity for modelling the behaviour of thin collagenous membranes. *Proc. Appl. Math. Mech.* **16**, 107–108 (2016).

18. Baughman, R. H., Stafström, S., Cui, C. & Dantas, S. O. Materials with negative compressibilities in one or more dimensions. *Science* **279**, 1522–1524 (1998).

19. Purohit, P. K., Litvinov, R. I., Brown, A. E. X., Discher, D. E. & Weisel, J. W. Protein unfolding accounts for the unusual mechanical behavior of fibrin networks. *Acta Biomater.* **7**, 2374–2383 (2011).

20. Demiray, H., Weizsäcker, H. W., Pascale, K. & Erbay, H. A. A stress-strain relation for a rat abdominal aorta. *J. Biomech.* **21**, 369–374 (1988).

21. Van Der Sman, R. G. M. Hyperelastic models for hydration of cellular tissue. *Soft Matter* **11**, 7579–7591 (2015).

22. Holzapfel, G. A. *Nonlinear Solid Mechanics: A Continuum Approach for Engineering.* John Wiley & Sons, Chichester (2000).

23. Schröder, J. & Neff, P. Invariant formulation of hyperelastic transverse isotropy based on polyconvex free energy functions. *Int. J. Solids Struct.* **40**, 401–445 (2003).

24. Ehret, A. E. & Itskov, M. A polyconvex hyperelastic model for fiber-reinforced materials in application to soft tissues. *J. Mater. Sci.* **42**, 8853–8863 (2007).

25. Janmey, P. A. et al. Negative normal stress in semiflexible biopolymer gels. *Nat. Mater.* **6**, 48–51 (2007).

26. Mihai, L. A. & Goriely, A. Positive or negative Poynting effect? the role of adscititious inequalities in hyperelastic materials. *Proc. R. Soc. A* **467**, 3633–3646 (2011).

27. Mauri, A. et al. Deformation mechanisms of human amnion: Quantitative studies based on second harmonic generation microscopy. *J. Biomech.* **48**, 1606–1613 (2015).

28. Hopf, R. et al. Experimental and theoretical analyses of the age-dependent large-strain behavior of Sylgard 184 (10:1) silicone elastomer. *J. Mech. Behav. Biomed. Mater.* **60**, 425–437 (2016).
