# Supplementary material for: Inverse poroelasticity as a fundamental mechanism in biomechanics and mechanobiology
Source: Nat Commun. 2017 Oct 17;8:1002. doi: 10.1038/s41467-017-00801-3 (PMC5714996; doi:10.1038/s41467-017-00801-3)
Supplement: Supplementary file 2 — Description of Additional Supplementary Information [file 41467_2017_801_MOESM2_ESM.pdf]

Description of Additional Supplementary Files

File Name: Supplementary Movie 1
Description: Sequence of multiphoton microscopy images of the near-field of a defect in a human amniotic membrane specimen that is elongated by 6 mm in steps of 0.5 mm. The densification of collagen at the defect site is visible as an increasing intensity of the SHG signal (green).
